# The effectiveness of a suicide prevention app for indigenous Australian youths: study protocol for a randomized controlled trial

**DOI:** 10.1186/1745-6215-14-396

**Published:** 2013-11-20

**Authors:** Fiona L Shand, Rebecca Ridani, Joe Tighe, Helen Christensen

**Affiliations:** 1 Black Dog Institute, University of New South Wales, Sydney, Australia; 2 Alive & Kicking Goals!, Broome, Australia

**Keywords:** App, Indigenous, Self-help, Suicide prevention

## Abstract

**Background:**

Indigenous Australian youth (aged 15 to 34) have up to four times the risk of suicide compared with their non-Indigenous counterparts. Barriers to help-seeking include shame, feared loss of autonomy and negative attitudes towards healthcare providers. The use of mobile devices and apps continues to rise amongst young people, thus presenting opportunities to utilize these aids in overcoming help-seeking barriers. Apps have been shown to assist in several health-related areas, including weight loss and smoking cessation, although no apps have as yet been evaluated for suicide prevention. Moreover, there is a lack of research that scientifically evaluates suicide prevention interventions within Indigenous communities.

**Methods/Design:**

In this study, a recently developed self-help app will be evaluated in a randomized controlled trial. The intervention is based on acceptance and commitment therapy and mindfulness-based cognitive behavioural therapy. It is aimed at participants who have suicidal thoughts but who are not actively suicidal. In total, 150 participants will be randomly allocated to the intervention-condition (*N* = 75) or to the wait-list control condition (*N* = 75). Questionnaires will be completed at baseline, post-test and 6 weeks follow-up. The primary outcome measure is a reduction in frequency and intensity of suicidal thoughts. Secondary outcome measures are the reduction of depression, anxiety and impulsivity.

**Discussion:**

This study is the first to evaluate the effectiveness of a self-help app for suicidal thoughts amongst young Indigenous people. Several limitations and strengths of the design are discussed.

**Trial registration:**

Australian New Zealand Clinical Trials Registry (ANZCTR): ACTRN12613000104752.

## Background

Suicide is the leading cause of death for Australians aged 15 to 34. The suicide rate for Indigenous people has increased dramatically over the past 30 years despite decreases in that for the general population. Overall, the suicide rate for Indigenous populations is twice that for non-Indigenous people with Indigenous youth being most at risk [1]. Compared with their non-Indigenous counterparts, Indigenous youth aged 15 to 24 have four times the risk of suicide, with those aged 25 to 34 having almost three times the risk [2].

Help-seeking amongst Indigenous populations is considerably lower than in the general community. Only 14% of Indigenous people who had a diagnosable mental illness at the time of suicide sought prior mental health treatment [3]. Lack of anonymity is problematic where individuals are part of a closely interwoven community and health workers are known to the help-seeking individual. Shame, stigma and the need to maintain esteem within the community are major barriers to help-seeking, as are cost (service or transport), service availability and service suitability [4].

Anonymous support may overcome some of these obstacles, and although the internet has gone some way to addressing help-seeking barriers in some communities, portable devices and apps may provide a more accessible means of support. The use of portable mobile devices (including smartphones and tablets) is increasing rapidly amongst youth. In mid-2012 more than 1.4 million apps were available on the market, some of which aimed to help people with their mental health and wellbeing or aid in the prevention of suicide. However, most have not been evaluated to determine their effectiveness.

Thirty-four per cent of people experiencing suicidal ideation go on to make a plan, and 29% attempt suicide [5]. This suggests that suicide prevention efforts need to be aimed at those with suicidal ideation. There is evidence that cognitive behavioural therapy, dialectical behaviour therapy and other cognitive interventions (that is, schema focused and problem solving therapies) are effective in reducing self-harm and suicidal behaviours [6,7]. Mindfulness-based cognitive therapy has also shown promising results in reducing suicidal ideation [8].

The current intervention uses acceptance and commitment therapy, the ‘third wave’ of cognitive behavioural therapy [9]. Although acceptance and commitment therapy is rigorously behavioural and based on scientific validity of human cognition, it also addresses issues of spirituality, values and self, components that have been identified as crucial to successful indigenous suicide prevention strategies. The goal of acceptance and commitment therapy is to assist individuals to cease struggling against their internal experiences, such as thoughts, feelings, memories and sensations, and help them move in valued directions by implementing effective behavioural changes. Acceptance and commitment therapy has been found to be efficacious in a number of mental health domains, including depression [10], anxiety [11] and stress [12]. It has demonstrated effect sizes similar to that of traditional cognitive behavioural therapy and superior effects to control conditions for a range of mental and physical health disorders [13].

Emerging evidence suggests that online intervention to prevent suicide is feasible and effective [14,15]. An international systematic review of suicide prevention for Indigenous populations identified only three studies based in Australia, none of which were controlled trials [16]. Given the prevalence of suicide amongst this community, there is an urgent need for feasible, acceptable interventions with evidence of effectiveness.

This pilot study has several aims including: assessing whether the app can reduce suicidal thinking, psychological distress or impulsivity; evaluating the acceptability of the app to young Indigenous people; assessing the feasibility of the recruitment, screening and randomization methods; examining uptake and adherence to the app; and evaluating the safety, security and clinical support protocols.

## Methods/Design

### Design

This study is a randomized controlled trial comparing a self-help app intervention with a wait-list control condition. The intervention is aimed at persons with suicidal thoughts but without active suicidal intent. Participants in the control condition will have access to the intervention after the experimental group has finished (that is, after six weeks). Assessments will take place at baseline, immediately after intervention completion, and 6 weeks follow-up. This study was approved by the University of New South Wales Human Research Ethics Committee (protocol no. HC13025) the Western Australian Aboriginal Health Ethics Committee (protocol no. 486), and the Kimberley Aboriginal Health Planning Forum (protocol no. 2013–006).

### Sample size

A maximum of 150 participants will be recruited for the study. Power analysis for the primary outcome measure indicates that 98 participants are needed for a medium effect size. We anticipate that some participants will be lost to follow-up, lose their tablets or incur damage to them, thus the data that we obtain at the end of the trial is likely to come from fewer than the 150 registered participants.

### Eligibility criteria and screening

Eligibility to take part in the pilot is determined in a stepwise screening process. To be eligible, participants must be: aged 18 to 30; able to attend two or three face-to-face sessions in Armidale, Sydney or Broome; consent to be asked questions of a personal nature; not have been diagnosed with a psychotic disorder such as schizophrenia; have had suicidal thoughts in the past two weeks; not be severely suicidal; and be willing to make contact with the Suicide Call Back Service.

Participants who meet the inclusion criteria will be administered the Kessler Psychological Distress Scale (K10) [17] and Patient Health Questionnaire-9 (PHQ-9) [18]. Participants are excluded from the study if they answer, ‘Not at all’, to item 9 on the PHQ-9, which asks participants to categorize how frequently they have had ‘Thoughts that you would be better off dead or of hurting yourself in some way’, over the previous fortnight.

Individuals are then asked several suicide intent questions. Those who indicate immediate intent to take their life and have a plan to do so are excluded from the study and receive Applied Suicide Intervention Skills Training. If their intent to suicide is reduced at the end of this intervention, they are given referral information to the Suicide Call Back Service. Alternatively, if the participant still expresses intent to suicide, their local acute care team will be called, and their contact details will also be given to a clinical psychologist for follow-up within 72 hours. Eligible participants who have indicated distress (by scoring >19 on the PHQ-9 or >29 on the K10) will still be admitted to the study, but will be referred to support services.

### Inclusion procedure

**Figure 1:**
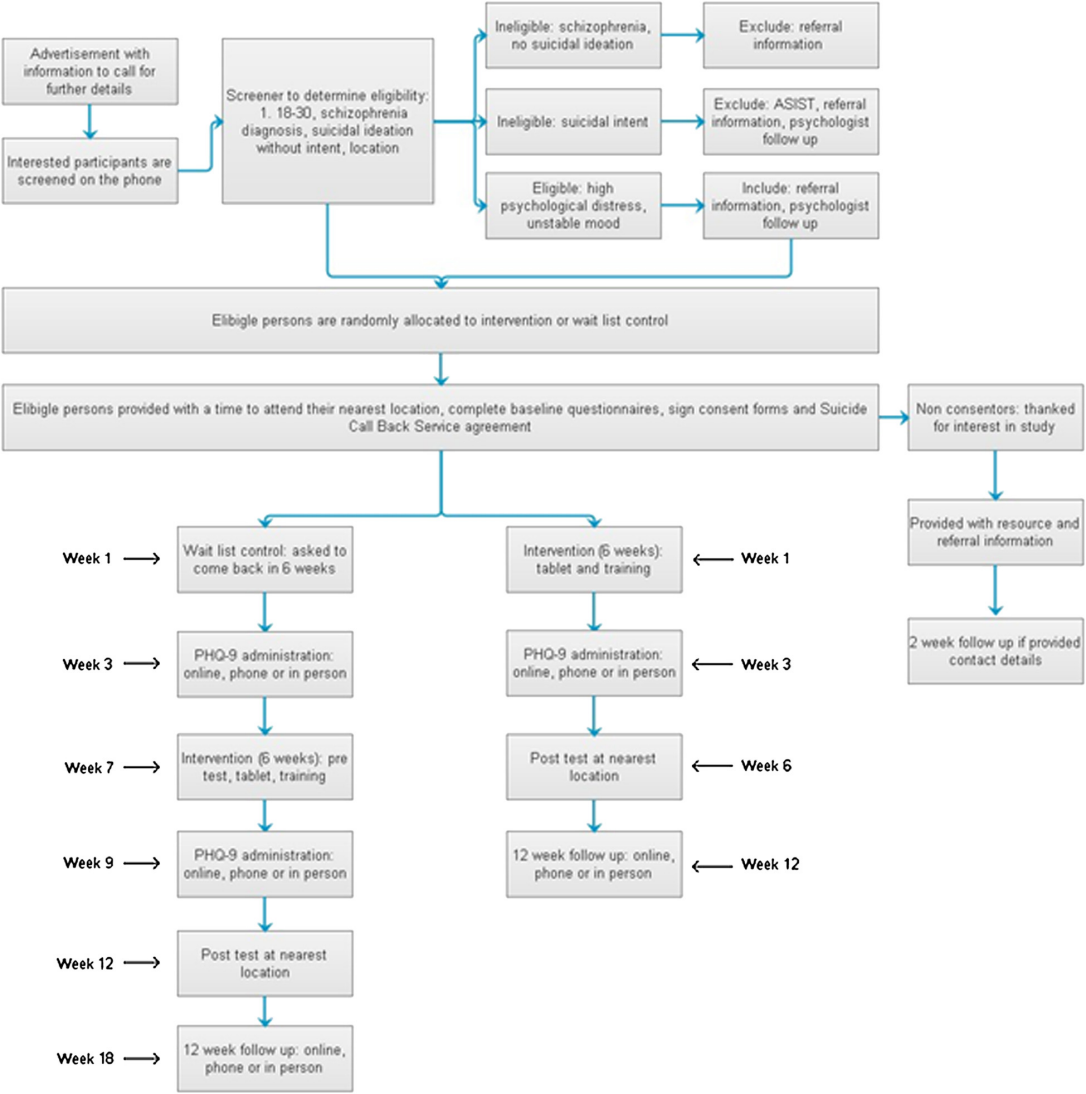
Study procedure.

Participants will be screened over the phone and those eligible will be given a time for their induction session. Participants in remote locations may opt to have the tablet couriered and to have their induction over the telephone. During induction, participants will read a participant information statement, sign a consent form and safety agreement, be administered baseline measures and complete a follow-up contact form so that the researchers can make contact with them or their closest contact for post-test and follow-up measures. Individuals in the intervention group will be provided with a tablet and basic training on how to use the app, as well as assistance in setting up their security access. Those in the wait-list control group will be asked to return to the same location after 7 weeks, at which point they will be given a tablet and basic app training. An overview of the process is given in Figure 1.

### Screening

#### Recruitment

Recruitment will take place via the Black Dog Institute (on its website and through their volunteer research registry), through advertisements on social networking sites, and through partner organizations *Alive and Kicking Goals!* in Broome and *BackTrack* in Armidale.

#### Randomization

After screening, participants are randomized to either the active or wait-list control group using block randomization (four per block).

#### Content of programme

The self-paced app aims to help participants decrease the frequency and intensity of their suicidal thoughts via the sequential completion of three modules, aimed at addressing thoughts, emotions and values. Participants are able to track their progress through a personalized dashboard. To cater for participants with low literacy levels, all text is accompanied by voice recordings. Although not intended as a crisis intervention tool, the app displays emergency contact information for several 24 hour helplines.

##### Module 1

Participants identify thoughts they may be experiencing and group them based on the frequency at which they occur. This is done by ‘swiping’ the thought down the screen into a basket. The thought that bothers them the most is then selected, and accompanying feelings and behaviours are identified, followed by psychoeducation, highlighting the links between thoughts, feelings and behaviours. Finally, participants are given strategies to help them defuse from their thoughts.

##### Module 2

Participants are taught to regulate their emotions through several strategies: mindfulness, which encourages participants to maintain non-judgemental contact with their psychological and environmental experiences as they occur; acceptance, which presents participants with an alternative to experiential avoidance and helps them to be aware of their internal experiences without the need to control them; and self-soothing activities that participants can engage in, including those that fall within social (for example, calling a friend), active (for example, going for a walk) and cultural (for example, spending time in nature) categories.

##### Module 3

Participants are taught to identify values that they would like to stand for, and asked to set small, achievable goals to help them live by these values. Rather than being end goals in themselves, values are characteristics that an individual cultivates. Examples include compassion, kindness and courage. At the end of this module, participants are given an action plan, based on their answers to the differing activities throughout the app.

### Data collection

Use of the app (that is, login and logout times, activities completed, time spent in each section, answers to self-assessment questions) will be tracked within the device, and downloaded to secure servers whenever internet connectivity is established. The app will require a security pin for access and any data stored on the device will be encrypted and hidden within the app coding. Answers to self-assessment questions will be coded so that they are unintelligible to anyone not connected with the study.

### Assessments

Baseline questionnaires will consist of items relating to demographics, help-seeking, psychological distress, depression, suicidality and impulsivity (Table 1). Measures will also be administered immediately after participants complete the interventions, and at 6 weeks follow-up. The Patient Health Questionnaire 9 (PHQ-9) is the depression scale of the Patient Health Questionnaire, and consists of nine items, each scoring from 0 (not at all) to 3 (nearly every day). Participants are asked to rate how problematic the items have been for them over the previous fortnight. Overall scores range from 0 to 27 with 0 to 4 representing no or minimal depression, 5 to 9 representing mild depression, 10 to 14 moderate depression, 15 to 19 moderately severe depression, and 20 to 27 severe depression [18].

**Table 1:** Overview of measures

**Variable**	**Test measure**	**Screener**	**Brief assessment**	**Baseline**	**Within app**	**Post-test**	**Follow-up**
Screener, part 1	Questions	X					
Psychological distress	K10	X		X		X	X
Depression	PHQ-9	X	X	X		X	X
Suicide intent	5 questions	X					
Demographics	Questions			X			
Mental health service utilization	Client Service Receipt Inventory			X			
Impulsivity	BIS-11			X		X	X
Suicidal ideation	DSI-SS			X		X	X
Suicidal ideation and plan	2 questions				X		

BIS-11, Barratt Impulsivity Scale; DSI-SS, Depressive Symptom Inventory – Suicidality Subscale; K10, Kessler Psychological Distress Scale; PHQ-9, Patient Health Questionnaire 9*.*

The Kessler Psychological Distress Scale (K10) contains ten items rated on their occurrence over the previous 4 weeks from 1 (none of the time) to 5 (all of the time). Total scores range from 10 to 50 with psychological distress being categorized as follows: those scoring 1 to 20 are likely to be well; those scoring 20 to 24 are likely to have a mild mental disorder; those scoring 25 to 29 are likely to have a moderately severe mental disorder; those scoring 30 and above are likely to have a severe mental disorder [17]. The scale has been used across several populations [19] and is a valid and reliable instrument sensitive to the detection of mental health conditions within the population [20].

The Barratt Impulsivity Scale (BIS-11) is the most widely used instrument to assess the personality/behavioural construct of impulsivity within individuals [21]. The 30-item questionnaire asks respondents to select how frequently they engage in common impulsive and non-impulsive behaviour. Responses are coded on a four-point scale from 1 (rarely or never) to 4 (almost always or always), with higher scores indicating greater impulsivity. The scale has good validity with internal consistency coefficients ranging from 0.79 to 0.83 and has been used with undergraduate students, substance abuse patients, prison inmates and general psychiatric patients [21].

The Depressive Symptom Inventory – Suicidality Subscale (DSI-SS) is a four-item self-report questionnaire designed to identify the frequency and intensity of suicidal ideation in the previous weeks [22]. Total inventory scores range from 0 to 12, with each answer being scored 0 to 3. Higher scores indicate greater severity of suicidal ideation. The four questions assess frequency of suicidal ideation, development of a suicide plan, an inability to control suicidal thoughts, and suicidal impulses. The scale has good reliability and validity [23,24] specifically amongst a group of 15 to 24-year-old general practice patients [25].

The Client Service Receipt Inventory is used in mental health service evaluations and collects retrospective information about the interviewee’s use of health and social care services, accommodation and income. It can be tailored to suit the data requirements, thus the following domains will be captured within this study: GP consultations, practice nurse visits, use of hospital services for physical or mental health problems, mental health helpline contacts, psychiatric crisis support team contacts, social worker contacts, counselling contacts, therapy contacts, self-help groups contact and psychiatrist contacts [26,27].

### Wait-list control group

The wait-list control group commence the programme six weeks after being administered the baseline questionnaires.

### Safety of participants

The app is a self-help intervention that is fully automated; although participants will be experiencing suicidal thoughts, they will be supported by 24 hour telephone crisis lines, including Lifeline and Kids Helpline. Prior to participating in the study, participants will be asked to sign a safety agreement that encourages them to call the Suicide Call Back Service if they feel distressed at any time during the study duration. Each participant will be contacted via email or telephone three weeks into the intervention period and during the wait-list period for a brief assessment.

#### Background to safety procedure

The first randomized controlled trial of an online suicide prevention programme was run in the Netherlands, with no suicides recorded for any of the 236 participants. Thus there was no indication that the programme resulted in adverse effects on the participants. The current pilot prepares for the first randomized controlled trial of an intervention with a similar population (that is, those experiencing suicidal thoughts).

#### Emergency help within the app

At the start of each module, participants will be asked a series of statements, one of which includes, ‘I am thinking of taking my own life.’ If a participant responds, ‘True’, to this statement, they are then asked whether the statement, ‘I’ve made plans to take my own life’, is true or not. An answer of, ‘True’, to this question triggers the emergency help page, which encourages the participant to immediately call one of three 24-hour crisis support lines: Lifeline, Kids Helpline, and emergency services. This emergency help page is accessible from anywhere within the app via a help button.

### Statistical analyses

To test the hypothesis that the self-help intervention is superior to the control condition, the analysis will be conducted on an intention-to-treat basis following the pertinent BMJ & Consort guidelines. Missing observations at follow-up will be imputed by regression imputation or multiple imputations, stratified for predictors of outcome and loss to follow-up. Relative improvements in frequency and intensity of suicidal thoughts for the experimental group in comparison with the control group will be calculated using Cohen’s *d*. For this confirmatory analysis the primary outcome measure is used (the DSI-SS). For the analyses of the secondary outcome measures, a Bonferroni correction will be applied to control the overall Type I error rate.

## Discussion

This paper describes the study protocol for a pilot randomized controlled trial of an app for suicide prevention in young Indigenous people. To our knowledge, this is the first trial of a mobile-based app for suicide prevention, and the first randomized controlled trial of suicide prevention with Indigenous Australians. Despite fears that focusing on suicidal ideation might give rise to further suicidal thoughts and even attempts, there is no evidence to validate this idea [15,28].

Several limitations apply to the study protocol of this self-help app. First, online interventions tend to have a high attrition rate. Dropout from the study can introduce a selection bias and therefore be a threat to validity. To minimize this, participants will be asked for additional contact details so that even if the intervention is not completed, follow-up assessments may still be collected. Further, each participant will be contacted via email or text message three weeks into the intervention or wait-list period for a brief assessment. A reminder to continue with the app will be provided at this point.

A further limitation is that none of the measures provides a psychiatric diagnosis. The intent is to keep the measures to a minimum in order to increase retention, as the research participants may find too many measures a barrier to participation. Some participants are also likely to have low literacy skills. Long, complex measures would be a significant problem for these participants. To overcome literacy problems, participants have the option of face-to-face or telephone assistance to complete the measures. Moreover, the app targets suicidal ideation irrespective of diagnosis and, as such, a measure of suicidality is a more appropriate primary outcome measure.

Researching suicide prevention is difficult; perhaps even more so within Indigenous communities. Because of this, little is known about what works with this population. While suicide rates have dropped amongst the general population over the last two decades, they remain stubbornly high in the Indigenous community [29]. This study will make a significant and unique contribution to our knowledge about suicide prevention and treating suicidal ideation.

## Trial status

Currently recruiting.

## Abbreviations

BIS-11: Barratt Impulsivity Scale; DSI-SS: Depressive Symptom Inventory – Suicidality Subscale; K10: Kessler Psychological Distress Scale; PHQ-9: Patient Health Questionnaire 9.

## Competing interests

The authors declare that they have no competing interests.

## Authors’ contributions

HC obtained funding for this study. All authors contributed substantially to the design of this study and the development of the intervention. FLS and RR drafted the manuscript. FLS, RR and JT will carry out the study. All authors contributed to further writing of the manuscript and read and approved the final manuscript.
